# Work-life balance practices and organizational cynicism: The mediating role of person-job fit

**DOI:** 10.3389/fpsyg.2022.979666

**Published:** 2022-09-14

**Authors:** Abdul Samad Kakar, Niel Kruger, Dilawar Khan Durrani, Muhammad Asif Khan, Natanya Meyer

**Affiliations:** ^1^Department of Management Sciences, University of Loralai, Loralai, Pakistan; ^2^DHET-NRF SARChI Entrepreneurship Education, University of Johannesburg, Johannesburg, South Africa; ^3^Institiute of Management Sciences, University of Balochistan, Quetta, Pakistan; ^4^Department of Commerce, Faculty of Management Sciences, University of Kotli Azad Jammu and Kashmir, Kotli, Pakistan; ^5^Department of Business Management, University of Johannesburg, Johannesburg, South Africa

**Keywords:** work-life balance (WLB) practices, organizational cynicism, person-job fit, PLS-SEM, higher order construct

## Abstract

This study aims to elaborate on how work-life balance (WLB) practices influence organizational cynicism (OC) through the mediation effects of person-job fit (PJF). We collected data from 331 nurses through a self-administered survey, and we tested our hypothesized model through partial least square structural equation modeling techniques using SmartPLS software. The findings revealed that WLB practices influenced OC negatively and PJF positively. We further found that PJF negatively influenced OC and mediated WLB practices’ effect on OC. These findings imply that nurses should be provided WLB practices to meet their job and home responsibilities and thus have a less cynical attitude toward the organization.

## Introduction

For the past 20 years, organizational cynicism (OC) defined as: employees’ negative attitude toward organization and management practices, has been a topic of intense debate among practitioners and researchers (Dean et al., 1998; Kökalan, 2019; Cicek et al., 2021; Atalay et al., 2022; Şen and Basım, 2022). For the most part, researchers have provided theoretical and empirical arguments for the impact of OC on essential work outcomes. These arguments have included:

• employees’ emotional exhaustion (Naseer et al., 2020),
• counterproductive and deviant work behaviors (Jiang et al., 2017; Naseer et al., 2020),
• job dissatisfaction (Dean et al., 1998; Kökalan, 2019),
• organizational commitment (Dean et al., 1998),
• turnover intentions (Munir et al., 2018; Sungur et al., 2019; Cicek et al., 2021),
• procrastination (Munir et al., 2018; Sungur et al., 2019), and
• employees’ extra-role and in-role performance (Jordan et al., 2007).

Organizational cynicism has also adversely affected employees’ productivity and performance (Rehan, 2017), and organizational citizenship behavior (Singh and Randhawa, 2022). Despite some clear-cut evidence of the adverse impact of OC on individual and organizational outcomes, research on factors that may cause employees’ (i.e., frontline healthcare nurses) negative attitudes toward organizations (i.e., OC) is limited.

One factor that has been acknowledged to promote positive work attitudes among employees is the provision and support of work-life balance (WLB) practices (Koon, 2022; Stamm et al., 2022). WLB practices are organizational practices that increase workers’ independence in balancing their work and family life. These practices include part-time work, telecommuting (i.e., working from home using technological devices), job sharing (when two or more employees share full-time job responsibilities voluntarily), flexible starting and finishing time, work leaves, and paid parental leaves (Felstead et al., 2002; Cegarra-Leiva et al., 2012; Parakandi and Behery, 2016).

Literature reveals that the use of WLB practices not only promotes harmony between employees’ personal and work-life but also engender positive attitudes, including job satisfaction (Dousin et al., 2021), organizational citizenship behavior (Cegarra-Leiva et al., 2012), and organization commitment (Onken-Menke et al., 2017; Koon, 2022). In addition, WLB practices have the potential to mitigate adverse work outcomes such as stress (Chiang et al., 2010), emotional exhaustion, burnout, turnover intention (Kakar et al., 2022), and work-family conflict (Onken-Menke et al., 2017). However, scholarly work on the role of WLB practices in discouraging frontline healthcare nurses’ cynical attitudes toward organizations is limited. Moreover, most studies on the relationship between WLB practices and OC have been conducted in the Western context, limiting their value in other contexts.

Furthermore, although many studies have well documented the direct association between WLB practices and work-related attitudes, the mediating mechanism through which WLB practices translate into OC has mainly been ignored. WLB practices essentially are the means through which employees’ knowledge, skills, and abilities (KSAs) are enhanced to meet their work and non-work responsibilities. WLB practices as organizational resources also fulfill employees’ needs and preferences. When employees believe that their KSAs are sufficient to meet the work responsibilities, and job resources are adequate to meet their needs and preferences, they are more likely to experience person-job fit (PJF) (Cable and DeRue, 2002; Chi et al., 2020; Kakar et al., 2020). PJF is one of the core components of the working environment and denotes the degree to which, on the one hand, individuals can address work/home responsibilities through their KSAs. On the other hand, individuals believe that the resources offered by the job meet their work and non-work needs and preferences. Besides, PJF is most frequently examined as a prominent predictor of important work-related attitudes (Cable and DeRue, 2002; Yu, 2016; Chi et al., 2020). Additionally, the literature suggests that organizational practices can predict PJF (e.g., training, development opportunities, orientation and socialization, performance appraisal, and compensation), and PJF, in turn, results in lower work attitudes (Boon et al., 2011; Kakar et al., 2019b; Uppal, 2020).

To address these shortcomings, this study aims to examine the influence of WLB practices on OC of the employees working in the healthcare industry of Pakistan- a non-westernized country, where research on OC and WLB practices is in the infancy stage. Furthermore, keeping in view the influence of organizational practices on PJF, and PJF’s resultant effect on work-related attitudes, we hope to add new insights to the literature by suggesting that WLB practices (e.g., telecommuting, job sharing, paid leaves, etc.) have the potential to manifest into OC through PJF.

In doing so, this study seeks to make four significant contributions. Firstly, to examine whether WLB practices discourage organizational cynicism and encourage PJF among frontline healthcare workers working in public sector hospitals in Balochistan, Pakistan. Secondly, to expand OC literature by exploring its relationship with WLB practices and PJF. Thirdly, to explore the mediating effect of PJF as a potential underlying mechanism through which WLB practices mitigate OC. Fourthly, to enhance understanding of how the mediating mechanism augments practitioners’ ability to: design and implement WLB strategies that enhance nurses’ capabilities to meet their work and family needs/demands; and, reduce nurses’ cynical attitudes toward organizations.

## Literature review

### Organizational cynicism

Cynicism refers to employees’ contemptuous, cynical, or negative attitudes toward an object, entity, organization, or group. Within the literature, different types of cynicism have been identified. For instance, personality cynicism represents the general mistrust of an individual toward his/her colleague (Abraham, 2000), while occupational cynicism reflects employees’ negative attitude toward stressful occupations (Ashforth and Humphrey, 1993). Societal cynicism is defined as the “negative view of human nature, a view that life produces unhappiness, that people exploit others, and a mistrust of social institutions”(Bond et al., 2004, p.533). Likewise, other scholars have defined OC as employees’ undesirable attitudes toward organizations and management practices (Cicek et al., 2021; Huang, 2022).

However, OC extends beyond cynicism as a multidimensional construct consisting of affective, cognitive, and behavioral dimensions (Dean et al., 1998). The cognitive dimension reflects employees’ belief that the organization with which they work is devoid of honesty and integrity. The affective dimension represents their negative attitude (e.g., feeling, emotions, anger, and disdain) toward the organization. Lastly, the behavioral dimension represents individual participation in unfavorable activities (e.g., engaging in negative word of mouth, procrastination, making malicious comments about an organization in front of others, time thief, arriving late, and leaving early etc.) in response to an individual cognitive belief and affective attitude toward organizations (Dean et al., 1998; Pfrombeck et al., 2020). This study conceptualizes and operationalizes OC as a higher-order construct with three dimensions, namely: affective, cognitive, and behavioral dimensions. This is because a higher-order construct reduces model complexities, improves model explanatory power and parsimony, and provides easier-to-understand relationships within the model (Becker et al., 2012; Hair et al., 2022).

### Person-job fit

Person-job fit represents the fit or match of an individual with job characteristics (Gander et al., 2020; Kakar et al., 2022). Cable and DeRue (2002) proposed two dimensions of PJF; namely demands-abilities-fit and needs-supplies fit. The former described the compatibility of a person’s KSAs with specific job requirements, while the latter denotes the match or fit between jobs’ resources and individuals’ needs and preferences (Yu, 2016; Pattanawit and Charoensukmongkol, 2022). As such, perceptions of PJF emanate from the congruence of personal attributes to the job characteristics.

In the literature, PJF has emerged as a significant predictor of essential work outcomes, including job satisfaction (Jiang et al., 2022), meaningfulness of work and work engagement (Guo and Hou, 2022), organizational citizenship behavior, organization commitment (Kristof-brown et al., 2005; Suwanti et al., 2018), and career success (Vogel and Feldman, 2009). PJF also reduces employees’ negative attitudes toward organizations, jobs, and people (Cable and DeRue, 2002; Vogel and Feldman, 2009; Yu, 2016; Uppal, 2020). Although PJF is an imperative concept in organizational studies, its antecedents and outcomes are relatively ignored in the literature (Harris and Pattie, 2020). Thus, this study examines WLB practices as an antecedent of PJF and organizational cynicism as its outcome.

## Hypotheses development

### Work-life balance practices and organization cynicism

It is worthwhile to discuss the concept of WLB practices before explaining its association with OC and PJF. WLB practices are formal and informal practices that simultaneously enhance individual autonomy in managing their work-life and family lives. Literature reveals that the use of WLB practices, on the one hand, engenders positive attitudes among employees (Onken-Menke et al., 2017; Koon, 2022), while on the other hand, mitigating negative work outcomes such as stress (Allen, 2001), burnout, emotional exhaustion (Deery and Jago, 2015; Padilla and Thompson, 2016). However, studies on the role of WLB practices in reducing nurses’ cynical attitudes are rare.

We predict that employees’ perception of WLB practices will influence their OC. That is, employees’ positive perception of WLB practices will decrease nurses’ OC, whereas their negative perception of WLB practices will result in an increased cynical attitude toward organizations. This prediction is based on Social Exchange Theory (SET) and related literature.

The core assumption of SET is that individuals feel obliged to reciprocate any good deeds on the part of the organization with desirable attitudes and behaviors. For example, when employees perceive that their organizations provide them with resources (e.g., WLB practices) that may benefit them, they tend to reciprocate by developing and displaying attitudes that are advantageous to organizations (Kuvaas, 2008; Kakar et al., 2022). On the other hand, employees who consider that their organizations do not provide work-life related practices, (e.g., flexible working hours, paid leaves, Flexi timing, and child-care benefits), will experience a high level of stress (Chiang et al., 2010), emotional exhaustion (Deery and Jago, 2015; Koon, 2022), and burnout (Padilla and Thompson, 2016); and therefore become more cynical toward their organizations (Chiaburu et al., 2013; Kim et al., 2019). To confirm this point, scholars have noted that providing employees with WLB practices will make them more competitive and autonomous in managing family and work-life, while organizations will find workers satisfied with the job and committed to the organization (Koon, 2022). The provision of WLB practices also sends signals to workers that they are being cared for and valued by the organization (Kakar et al., 2019a). Previous empirical studies also support the notion that WLB practices reduce negative attitudes or cynical attitudes among workers (Deery and Jago, 2015; Kakar et al., 2022; Koon, 2022). Thus, following SET and related literature, when frontline healthcare nurses believe that the organization they work for cares about them and values them, they will reciprocate by becoming less cynical toward the organization. Therefore, we propose that:

H1: Frontline healthcare nurses’ perceptions of WLB practices relate negatively to OC.

### Work-life balance practices and person-job fit

We also expect that nurses’ perceptions of WLB practices positively affect PJF. That is, high perceptions of WLB practices will increase nurses’ fit with their job. In contrast, a lack of WLB practices will likely decrease their compatibility with the job. Prior research has shown that PJF is dynamic and can change over time (Ferris et al., 1985). WLB practices can play a vital role in changing a person’s fit with his or her job. For instance, WLB practices such as telecommuting, job sharing, flexi timing, and child-care benefits, enhance individuals’ abilities to meet their work/home requirements. On the other hand, these practices signal to the employees that the organization they work for is caring, kind, and willing to meet their needs and preferences. Thus, when employees believe that the organizational resources meet their desires, and their abilities are compatible with the job requirements, they perceive a strong fit with the job (Cable and DeRue, 2002). Moreover, organizations have used WLB practices as strategic HRM practices to reduce employees’ work-life conflict (Davis et al., 2014) and improve their “Goodness of fit” between work and family life (Newman and Mathews, 1999). Thus, based on the preceding discussions, we propose that offering nurses WLB practices is likely to increase their fit with the job.

H2: WLB practices relate positively to PJF.

### Person-job fit and organizational cynicism

This study predicts that a person with a high level of PJF is less likely to have a cynical attitude toward organizations than a person low on PJF. This is because a person high on job fit believes that he/she has essential KSAs to perform a job, and the job has sufficient resources to meet his/her needs and preferences. From the social exchange perspective, when an employee perceives that the job resources are adequate for meeting job expectations, he/she feels obliged to reciprocate with a positive attitude, such as becoming less cynical toward the organization. It is proven that a person with a high job fit feels satisfied with the job (Vogel and Feldman, 2009), is motivated (Cable and DeRue, 2002), and experiences improvement in their well-being (Choi et al., 2017), and performs better (Boon et al., 2011). In contrast, people low on PJF are emotionally exhausted, physically burnt out, and always intend to switch over the job (Cable and DeRue, 2002; Samad et al., 2022). Thus, based on related literature and SET, we propose that:

H3: PJF relates negatively to OC.

### Work-life balance practices, person-job Fit, and organizational cynicism

Thus far, we have proposed that WLB practices will likely reduce OC (Hypothesis 1) and strengthen nurses’ fit with the job (Hypothesis 2). It was also proposed that PJF relates negatively to OC (Hypothesis 3). Thus it is logical to propose that PJF mediates the influence of WLB practices on OC.

Scholars have proposed, and there has been empirical support for the idea that PJF explains the underlying mechanism through which organizational practices influence essential work outcomes. For instance, Li et al. (2021) found that PJF mediated the influence of job crafting on job satisfaction. Boon et al. (2011) and Uppal (2020) found that PJF mediated the influence of certain HRM practices on job performance and turnover intention. By exploring PJF as a mediator between WLB practices and OC, this paper seeks to add new insight to the literature.

The notion that WLB practices relate to reduced OC indirectly through PJF, is explained in two ways. First, WLB practices can improve employees’ demand-ability fit—a dimension of PJF. For instance, the provision of WLB practices such as Flexi timing, flexible working hours, and autonomy, may reduce employees’ role ambiguity (Bulger and Fisher, 2012) and job requirements (Kakar et al., 2022), thereby increasing their abilities to fulfill job demands. For example, job sharing and telecommuting allow employees to have more control over the job (Nicklin et al., 2016), thus enhancing their capabilities to simultaneously manage work and family demands (Yao et al., 2017). Second, WLB practices can improve employees’ need-supply fit- the second dimension of PJF. For example, WLB practices initiatives such as: child-care, parental-care benefits, and paid time off, are job resources that reduce employees’ work-role conflict (Allen, 2001) and increase their participation in work and non-work domains. Furthermore, WLB practices provide employees with the resources that meet their needs and preferences (Kakar et al., 2019a), thus promoting the sense of need-supply fit.

Combining these theoretical arguments, we propose that WLB practices relate positively to PJF. Prior research already contends a significant and negative association between PJF and negative work attitude (Kristof-brown et al., 2005; Vogel and Feldman, 2009; Yu, 2016). These arguments suggest that WLB practices have both a direct (Hypothesis 1) and indirect influence on OC, and PJF mediates the indirect effect. Thus, we propose that:

H4: PJF mediates the influence of WLB practices on OC.

**FIGURE 1:**
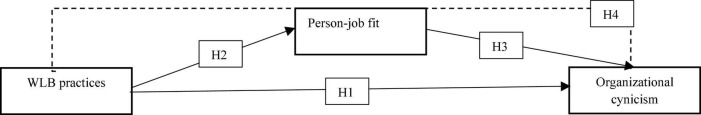
Conceptual model.

This relationship is presented in the model illustrated in Figure 1.

## Methodology

### Sample

This study aimed to examine the direct and indirect effect of WLB practices on frontline healthcare workers OC; therefore, the study’s target population were healthcare service providers (i.e., nurses) of three public sector hospitals in Balochistan, Pakistan. Balochistan is the largest but least developed (in terms of the healthcare system, education, infrastructure, and other facilities) province of Pakistan. Besides, the healthcare system in Balochistan is abysmal. For instance, healthcare employees (e.g., nurses and doctors) are deprived of basic life-saving equipment in hospitals. Their salaries and financial benefits are comparatively low as compared to other professions. In addition, organizational politics, workplace bullying, long working hours, and demanding and challenging jobs are other areas of concern that have exacerbated the healthcare working environment. Thus, working in such an environment not only reduces employees’ productivity and performance but also causes their cynical attitudes toward organizations. Therefore, we argue that investigating nurses’ cynicism toward the organization and how WLB practice can mitigate it is an interesting area of research and a need of time. Therefore, the target population of the study were nurses. Further, we conducted a cross-sectional and non-experimental self-administered survey among healthcare nurses for data collection. The data was obtained from the healthcare nurses that were conveniently and readily available for participation in the research (i.e., using convenience sampling, a non-probability sampling).

The study’s sample size was calculated using Faul et al. (2007) power analysis in G*Power 3.1 software. According to Hair et al. (2017), power analysis is commonly used for sample size detection in PLS-SEM literature. Using Hair et al. (2017) recommended 80% statistical power, 0.05 significance level, and a minimum 0.10 *R*^2^ value, we estimated that the required minimum sample size for this research is 84.

Initially, 450 questionnaires were distributed personally to the healthcare nurses, of which 351 were returned, indicating an initial response rate of 78%. Of 351, 20 cases were deleted based on suspicious responses and missing values. The elimination of 20 cases resulted in a usable response of 331 cases, representing an effective response rate of 73.55%. Out of 331 participants, 62.53% (*n* = 207) were female, and 37.47% (*n* = 124) were male. The participants’ age ranged from 21 to 58 years. Out of the total participants, 45.92% (*n* = 152) had work experience of 1–5 years, 21.65% (*n* = 76) had a working tenure of 6–10 years, 11.11% (*n* = 39) had worked for their organization from 11 to 15, and the remaining 18.23% (*n* = 64) had a working experience of more than 16 years. 87.31% (*n* = 289) participants had attended postgraduate education, while the remaining 12.68% (*n* = 42) had a Master of Philosophy degree.

### Measures

All constructs were measured with well-validated scales adapted/adopted from the literature. The scales for the measurement of WLB practices were adapted from Parakandi and Behery (2016). Nurses were asked about their perceptions of the availability of the WLB practices: Flexi-time, job sharing, part-time work, compressed workweek, telecommuting, child-care, parental-care benefits, and leave provisions. PJF was measured using Cable and DeRue (2002) six items scale of overall PJF. Similarly, 12 items were used for the measurement of OC. The items were adopted from the work of Durrah et al. (2019). Of the 12 items, 4 were used for the cognitive dimension of cynicism. The affective dimension of cynicism was measured with 4 items from Dean et al. (1998) questionnaires. Finally, 4 items were used for the measurement of behavioral cynicism.

All scales were rated on a five-point Likert scale: “1 = Strongly Disagree and 5 = Strongly Agree.”

## Results and method

Table 1 depicts the study’s descriptive statistics, including means, standard deviation (SD), and correlations among constructs. The mean for all constructs of the study ranged from 3.01 to 3.14. The correlation between WLB practices and the OC was negative and significant (−0.623**). Likewise, the correlation of WLB practices with PJF was positive (0.659**), while the PJF correlation with OC was negative and significant (−0.663**).

**TABLE 1:** Descriptive statistics.

Constructs	Mean	Std. deviation	1	2	3
(1) WLB Practices	3.011	0.88	–	0.659**	−0.623**
(2) Person-job fit	3.072	1.00		–	−0.663**
(3) OC	3.144	0.79			–

*N* = 331; **Significant 0.05; WLB, work-life balance practices; OC, organizational cynicism.

The model of the study was assessed using structural equation modeling (SEM). SEM uses both latent (unobservable) constructs, which are theoretical concepts and cannot be measured directly, and observable indicators/items, which are used to measure latent constructs (e.g., “scaling” a construct. Following the recommendations of Hair et al. (2022), we opted for the partial least square (PLS) SEM approach. This is because, PLS-SEM is not sensitive to data normality and sample size, and can avoid indeterminacy problems of the covariance-based SEM approach (Jravis et al., 2003). Furthermore, we used SmartPLS 3.2.8 software to analyze the model.

Following Hair et al. (2019): the model shown in Figure 1 was evaluated in two stages. In stage one, the measurement or outer model was assessed for constructs’ internal consistency and validity. In stage two, the structural model was assessed for hypothesis testing after confirming the constructs’ reliability and validity.

### Stage one: Measurement model

The measurement model valuation assures that the latent constructs of the study are truly represented by their observable items/indicators. Since this study has higher-order constructs (i.e., OC) and lowers order constructs (i.e., WLB practices, PJF, and dimensions of OC), therefore, following Becker et al. (2012), we first calculated the reliabilities and validities of lower-order constructs. Thereafter, we analyzed the reliability and validity of the higher-order construct (i.e., OC). In other words, we measured whether items consistently measure what they are supposed to measure.

**FIGURE 2:**
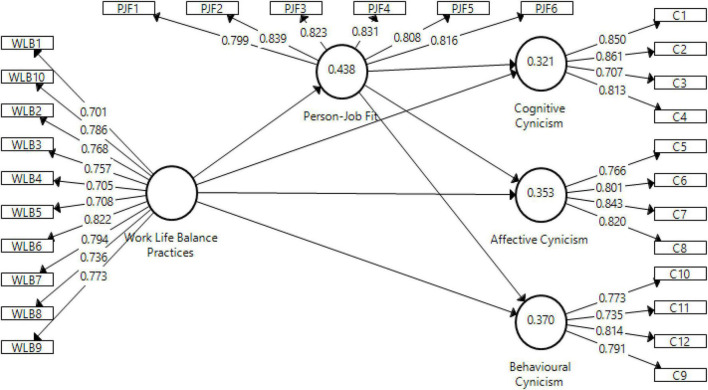
Measurement model.

The reliabilities of the lower-order constructs’ items were assessed through factor loadings (FLs). FLs refer to the relationship between latent constructs and observable indicators. According to Bryne (2010), an item is presumed to be reliable if its FLs values are ≥0.60. The results in Table 2 and Figure 2 show that all construct FLs are ≥0.60. To assess the constructs’ reliability, we calculated the Cronbach Alpha (CA) values and the composite reliabilities (CRs). CA and CRs measure the internal consistency reliability of constructs. A construct is presumed to be reliable if it has CA and CRs values ≥0.70 (Hair et al., 2019). As shown in Table 2, all constructs CA and CRs values exceeded the recommended threshold, thus confirming the internal consistency reliability of the lower-order constructs.

**TABLE 2:** Measurement properties of lower-order constructs.

Lower-order constructs	Items	Factor loadings	CA	CR	AVE
Cognitive cynicism	C1	0.850	0.823	0.884	0.656
	C2	0.861			
	C3	0.707			
	C4	0.813			
Affective cynicism	C5	0.766	0.822	0.882	0.653
	C6	0.801			
	C7	0.843			
	C8	0.820			
Behavioral cynicism	C9	0.791	0.784	0.860	0.606
	C10	0.773			
	C11	0.735			
	C12	0.814			
Person-job fit	PJF1	0.799	0.902	0.925	0.672
	PJF2	0.839			
	PJF3	0.823			
	PJF4	0.831			
	PJF5	0.808			
	PJF6	0.816			
Work-life balance practices	WLB1	0.701	0.916	0.930	0.572
	WLB2	0.786			
	WLB3	0.768			
	WLB4	0.757			
	WLB5	0.705			
	WLB6	0.708			
	WLB7	0.822			
	WLB8	0.794			
	WLB9	0.736			
	WLB10	0.773			

CA, cronbach alpha; CR, composite reliability; AVE, average variance extracted.

Further, the lower-order constructs’ validities were evaluated using convergent and discriminate validity. The former assumes that a set of observable items represent the underlying constructs, and can be measured by Average Variance Extracted (AVE). AVE represents the amount of variance in a construct that is explained by its indicator rather than measurement error. According to Fornell and Larcker (1981), a construct is convergently valid if 50% (AVE ≥0.50) of its variance is caused by its indicators rather than indicators of other constructs in the study. The findings presented in Table 2 portray that all constructs’ AVE values exceed the 0.50 threshold, thus affirming that all the lower-order constructs are convergently valid.

Discriminate validity, which represents the distinctiveness of a construct from other constructs in the model was estimated through two tests. The first test assumes that an underlying construct should explain more variance with its indicators than with other constructs’ indicators (Fornell and Larcker, 1981). Simply put, a construct’s correlation with other constructs should be lower than its square root of AVE (Table 3).

**TABLE 3:** Fornell and Larcker criteria.

Constructs	1	2	3	PJF	WLBP
1. AC	0.808				
2. BC	0.419	0.779			
3. CC	0.703	0.396	0.810		
4. PJF	−0.534	−0.596	−0.509	0.820	
5. WLBP	−0.548	−0.485	−0.523	0.662	0.756

AC, affective cynicism; BC, behavioral cynicism; CC, cognitive cynicism; PJF, person job fit; WLBP, work-life balance practices.

The second test conducted for the assessment of discriminate validity was Heterotrait-Monotrait Ratio (HTMT). HTMT is the “average of the hetero-trait-heteromethod correlations (i.e., the correlations of indicators across constructs measuring different phenomena), relative to the average of the monotrait-heteromethod correlations (i.e., the correlations of indicators within the same construct”) (Henseler et al., 2015, p. 122). According to Hair et al. (2019), a construct is discriminately valid if its HTMT values are less than 0.90. As shown in Table 4, all HTMT values are less than 0.90 thresholds, thus providing further support for discriminant validity.

**TABLE 4:** Heterotrait-Monotrait Ratio (HTMT).

Constructs	AC	BC	CC	PJF	WLBP
Affective cynicism (AC)					
Behavioral cynicism (BC)	0.514				
Cognitive cynicism (CS)	0.849	0.495			
Person-job fit (PJF)	0.619	0.703	0.591		
Work-life balance practices (WLBP)	0.631	0.554	0.597	0.725	–

AC, affective cynicism; BC, behavioral cynicism; CC, cognitive cynicism; PJF, person job fit; WLBP, work-life balance practices.

Finally, we estimated the higher-order construct (i.e., OC) measurement properties such as the significance and relevance of outer weights of dimensions of OC on OC as suggested by Becker et al. (2012) and Hair et al. (2017). For the significance of the outer weights, we used the bootstrapping procedure (5,000 subsamples). The findings (Table 5) showed that the outer weights of OC dimensions (i.e., lower-order constructs such as affective cynicism, behavioral cynicism, and cognitive cynicism) on OC (i.e., higher-order construct) were significant, thus confirming the validity of the OC as a higher-order construct.

**TABLE 5:** Outer weight of the dimensions of organizational cynicism (OC) on OC.

Constructs level		Weight	*t*-values	*p*-values
			
Higher-order construct	Lower-order constructs (OC dimensions)			
Organizational cynicism	Cognitive cynicism	0.29**	3.60	0.000
	Affective cynicism	0.38**	4.48	0.000
	Behavioral cynicism	0.54**	8.29	0.00

**Significant at 0.05 level based on 5,000 bootstraps.

### Stage two: Structural model assessment

After confirming the measurement model’s goodness of fit, we then analyzed the structural model. The structural model analysis estimates the strength and significance of the relationships depicted in Figure 1. In particular, we estimated the model’s significance of the path coefficient and *t*-statistics.

**FIGURE 3:**
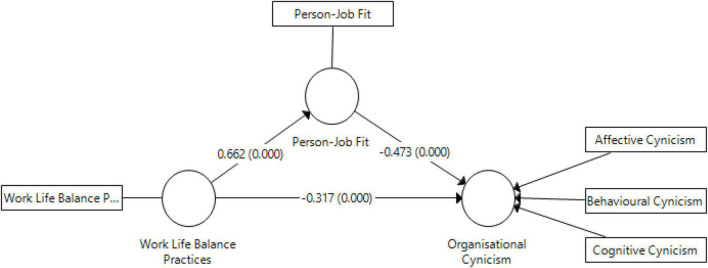
Structural model.

Concerning the path coefficients’ significance and strength, a bootstrapping procedure recommended by Hair et al. (2017) was used. The bootstrapping procedure is a commonly used non-parametric test that randomly draws several subsamples with replacements from the original data set. The path coefficients of the model were estimated with 5,000 subsample bootstrapping procedures. The results show that all hypotheses of the study were statistically significant, including the indirect effect of WLB practices on OC through PJF (see Table 6 and Figure 3).

**TABLE 6:** Structural model results.

Hypothesis	β	*T* statistics	*P*-values	Decision
Work-life balance practices – > Organizational cynicism	−0.317*	6.219	0.000	Supported
Work-life balance practices – > Person-job fit	0.662*	22.517	0.000	Supported
Person-job fit – > Organizational cynicism	−0.473*	9.858	0.000	Supported
Work-life balance practices – > Person-job fit – > Organizational cynicism	−0.313*	8.767	0.000	Supported

*Significant at *p* < 0.05.

### Mediation analysis

Prior to mediation analysis, certain conditions must be fulfilled (Hair et al., 2017). First, the direct effect of the independent variable (WLB practices) on dependent variables (OC) and mediator (PJF) must be significant. Second, the path coefficients between PJF and OC shall be significant. Third, the indirect effect of WLB practices (through PJF) on OC should be significant. Fourth, when a mediator is introduced in the structural model, the direct influence of WLB practice on OC shall decrease or become insignificant. As shown in Figure 3 and Table 6 the present study fulfilled all conditions of the mediation analysis.

After confirming the conditions of mediation analysis, we introduced PJF as a mediating variable between WLB practices and OC, and run the model using Preacher and Hayes (2008) bootstrapping procedure. The results obtained revealed that the path coefficient between WLB practices and OC lose significance, thus indicating that there is a full mediation.

## Discussion

This study’s first objective was to examine the direct effect of WLB practices on OC and PJF. Second, the study intended to explore whether nurses’ perception of PJF relates to OC. Third, the study explores the underlying mechanism (i.e., mediation) through which WLB practices influence OC. The results of data analysis confirmed that WLB practices significantly and negatively affected nurses’ cynical attitude toward organization. This finding implies that an organization may mitigate employees’ cynical attitudes toward the organization by providing WLB practices. For instance, with the provision of organizational practices (e.g., flexible working hours, paid parental leave, and telecommuting etc.), an organization may engender a sense of feeling among employees that their organization is supportive and caring. This perception of care and support will develop positive attitudes among them (Saufi et al., 2020; Kakar et al., 2022) and thus reduce their cynical attitudes toward organizations. The finding that WLB practices relate negatively to OC is coherent with the work of Dysvik et al. (2014) and Koon (2022). These authors state that WLB practices are essential to engender positive employee attitudes. For instance, when organizations implement WLB practices, employees become more satisfied and committed to the organization (Koon, 2022). In addition, a plethora of research has shown that WLB practices effectively repress negative attitudes and behaviors, such as stress, negative emotions, dissatisfaction, turnover intention, emotional exhaustion, and burnout (Chiang et al., 2010; Medina-Garrido et al., 2019; Kakar et al., 2022). Thus, in line with SET and related literature, this study has shown that with the provision of WLB practices, an organization can mitigate the cynical attitudes of the nurses toward their organizations. The findings also found that WLB practices are a significant predictor of PJF. This implies that an organization with the provision of WLB practices enhances nurses’ capabilities to meet the job responsibilities. For instance, providing leave, job sharing, and child-care benefits will enable nurses to meet their home responsibilities and thus allow them to focus more on work-related responsibilities. Besides, such practices will also fulfill their work and life-related needs and preferences. The finding that WLB practices relate to PJF is in line with the work of (Newman and Mathews, 1999; Boon et al., 2011; Kakar et al., 2022), who found that the provision of WLB practices enhances employees’ fit with the organization and job. Thus, WLB practices are part of strategic HRM practices that organizations may use to address employees’ work and life-related issues. The finding that WLB practices relate to PJF is also in line with numerous other studies that have shown the influence of organizational practices in strengthening employees’ fit with the job. For instance, Kakar et al. (2018), and Samad et al. (2022) found the positive influence of organizational practices on PJF. Overall, these studies suggest that the provisions of organizational practices (e.g., WLB practices) increase employees’ fit with the job for two reasons. First, these practices enable employees to develop more KSAs to meet job requirements. Second, organizational investment in WLB practices provides employees with sufficient resources that may meet their needs and desires.

The results also endorsed the negative relationship between PJF and OC. This finding implies that if employees’ attributes are compatible with the job characteristics, they are less likely to exhibit cynical attitudes toward organizations. In line with the present study, previous studies have also confirmed the direct influence of PJF on a variety of attitudinal variables (e.g., job satisfaction, organizational commitment, and intention to stay) (Cable and DeRue, 2002; Vogel and Feldman, 2009; Saufi et al., 2020; Kakar et al., 2022; Samad et al., 2022). These studies suggest that when employees observe that their KSAs are identical to the job requirements and job resources are adequate enough to meet their needs and preferences (i.e., PJF), then they exhibit positive attitudes. In contrast, in case of any incongruence between a person and job characteristics (i.e., poor PJF), negative attitudes tend to ensue. The finding is also parallel to the work of Allen (2001) and Onken-Menke et al. (2017), who suggested that provisions of work-life benefits reduce employees’ negative attitudes. Besides, the results also provide support to the SET assertion that employees reciprocate with positive attitudes when they receive benefits (e.g., work-life benefits) from organizations that satisfy their needs and desires.

In addition, this research has shown that PJF mediated the impact of WLB practices on OC. This finding suggests that when organizations invest in WLB practices, employees may believe these investments are intended to enhance their capabilities to meet the job requirements. Besides, the investment in WLB practices also signals to the frontline healthcare nurses that the organization they work for cares and is concerned about their needs. Thus, organizational investment in WLB practices results in PJF in two ways. Firstly, it strengthens nurses’ abilities to meet job requirements. Secondly, the resource provided by the organization in the form of WLB benefits fulfills their needs. In turn, PJF facilitated by WLB practices increases nurses’ satisfaction and commitment and reduces emotional exhaustion (Lu et al., 2012; Koon, 2022), thereby reducing their cynical attitudes toward organizations. In simple words, WLB practices lead to lower OC *via* enhancing the level of perceived PJF. The findings that PJF mediates WLB practices’ impact on turnover intention is also in line with other related studies (Kakar et al., 2022; Samad et al., 2022) that organization investment in organizational practices first strengthens employees’ fit with the job, which, in turn, mitigate their negative attitudes such turnover intention and cynicism.

## Implications of the study

This research has made several contributions to the body of knowledge. First, a significant contribution of this study lies in identifying a negative and important association between nurses’ perceptions of WLB practices and OC and of an indirect impact of WLB practices on OC through PJF. These findings provide insights into the evasive linkage between WLB practices and organizational and individual outcomes, an issue that scholars need to pay attention to improve our understanding of how WLB practices are linked to work outcomes. Another significant contribution of this study is the direct and positive influence of WLB practices on PJF, and PJF’s negative relationship with OC. To our understanding, we are not aware of a single study on the stated relationship in the available research.

In addition to theoretical contributions, this study provides important implications for the management of public sector hospitals. Like previous research, our study suggests that nurses’ negative attitude toward the organization is dynamic and can be changed with work-life balance provisions, organizational practices and organizational interventions. For instance, when organizations provide WLB practices, employees feel that the organization is caring and supportive of them. This perception of care will encourage employees to reduce their cynical attitude toward the organization. Besides, our data suggest that WLB practices (e.g., Flexi timing, telecommuting, child care, and parental-care benefits, etc.) also increase nurses’ abilities to meet their job requirements and fulfill their needs and preferences. Thus, for practitioners, to reduce nurses’ cynical attitudes toward organizations and enhance their perception of PJF, specific organizational practices that address their home and work responsibilities should be designed. For instance, telecommuting, job sharing, and Flexi timing will enable nurses to give sufficient time to their family and work. Besides, the provision of such practices will reduce their work role conflict, stress, burnout, and emotional exhaustion (Allen, 2001; Parakandi and Behery, 2016; Onken-Menke et al., 2017) caused by over timing and work pressure, thereby, consequently reducing their cynical attitude toward organizations. Furthermore, organizations may also provide family-related benefits (e.g., child-care and parental-care benefits) and family leave to address their needs and desires. Thus, the introduction of such practice will signal to the nurses that the organization cares about them and values them. In the norm of reciprocity, nurses will reciprocate this good treatment on the part of the organization with positive attitudes, such as lowering their cynical attitude toward the organization and strengthening their perception of fit with the job.

One significant contribution of this study was the partial mediating role of PJF between WLB practices and OC. This finding suggests that, on the one hand, that management can strengthen employees’ perception of fit with the job requirements and resources by offering WLB practices, and on the other hand, this perception of PJF can ultimately reduce OC. Thus, introducing WLB practices that improve PJF could lead to a reduced cynical attitude among nurses toward the organization. However, for this to happen, management must ensure that WLB practices are implemented, nurses are informed about the availability of WLB practices, and the accessibility of these practices is ensured. For instance, if an organization implement WLB practices but fail to inform employees about the provision of WLB practices, in such case, the WLB practices will produce the desired outcomes for the organization due to lack of communication. Hence, not only do organizations need to introduce WLB practices but they are also required to communicate about the benefits of WLB practices to their employees. The communication of such practices will reduce any misunderstanding between employees and employers and thus reduce employees cynical attitude toward the organization.

Nonetheless, PJF is partially explained by WLB practices. Management should keep in mind that other variables may also affect PJF. For instance, HRM practices (e.g., training, compensation, development, and growth opportunities (Kakar et al., 2019b; Uppal, 2020; Samad et al., 2022), and organizational support (Tseng and Yu, 2016) have an important effect on PJF. Similarly, long working hours (Merecz-Kot and Andysz, 2017) and work demands, among other factors, have a significant impact on PJF.

This study has potential implications for the government of Balochistan, Pakistan. It is suggested that the government not only introduce WLB practices (e.g., maternity leave, telecommuting, job sharing, and flexible working), but also ensure these practices are implemented and utilized by nurses. Additionally, the government must ensure that these practices are congruent with the needs and desires of the nurses. These are important suggestions, given that WLB practices in Pakistan’s organization is very poor and most employees work over 48 h per week.

## Study limitations

This study has several potential limitations. First, the causal relationships between WLB practices and OC cannot be conclusive because of the study’s cross-sectional nature. Thus, a longitudinal study is required to test the model. Second, findings of the may be biased because of the self-reported data. Third, PJF partially explained the indirect influence of WLB practices, hence, there is the probability of other factors that may explain the influence of WLB practices on OC. Thus, future researchers may test person-organization fit, person-supervisor fit, person-reform fit, WLB supportive culture, and employee attitudes (e.g., organization commitment, job satisfaction, stress, anxiety, and depression) as mediating variables between WLB practices and OC. Finally, the study respondents come from hospitals (public sector) in Pakistan; thus, the generalizability of findings should be interpreted with caution. Future researchers may test the model of the study among other professionals such as Doctors, educationists, tourism, manufacturing, and information technology sectors employees. This is because conducting studies in different contexts improves the generalizability of the study and reduces common method bias. We also encourage researchers to extend the body of knowledge by investigating WLB practices and OC with moderating variables such as job opportunity, organizational support, employability, work-life balance, work-life conflict, and organizational support.

## Data availability statement

The original contributions presented in this study are included in the article/supplementary material, further inquiries can be directed to the corresponding author.

## Author contributions

All authors listed have made a substantial, direct, and intellectual contribution to the work, and approved it for publication.
